# Ambient air pollutants are associated with morning serum cortisol in overweight and obese Latino youth in Los Angeles

**DOI:** 10.1186/s12940-021-00713-2

**Published:** 2021-04-08

**Authors:** C. M. Toledo-Corral, T. L. Alderete, M. M. Herting, R. Habre, A. K. Peterson, F. Lurmann, M. I. Goran, M. J. Weigensberg, F. D. Gilliland

**Affiliations:** 1 Department of Health Sciences, California State University Northridge, 18111 Nordhoff Street, Northridge, 91330 USA; 2 Department of Preventive Medicine, Environmental Health Division, University of Southern California, Keck School of Medicine, Los Angeles, USA; 3 Department of Integrative Physiology, University of Colorado at Boulder, Boulder, USA; 4 Sonoma Technology, Inc., Petaluma, USA; 5 Childrens Hospital Los Angeles, Los Angeles, USA; 6 Department of Pediatrics, University of Southern California, Keck School of Medicine, Los Angeles, USA

**Keywords:** Ambient air pollution, Ozone, Particulate matter, HPA-axis, Serum cortisol, Hispanic, Children

## Abstract

**Background:**

Hypothalamic-pituitary-adrenal (HPA)-axis dysfunction has been associated with a variety of mental health and cardio-metabolic disorders. While causal models of HPA-axis dysregulation have been largely focused on either pre-existing health conditions or psychosocial stress factors, recent evidence suggests a possible role for central nervous system activation via air pollutants, such as nitrogen dioxide (NO_2_), ozone (O_3_) and particulate matter (PM). Therefore, in an observational study of Latino youth, we investigated if monthly ambient NO_2_, O_3_, and PM with aerodynamic diameter ≤ 2.5 (PM_2.5_) exposure were associated with morning serum cortisol levels.

**Methods:**

In this cross-sectional study, morning serum cortisol level was assessed after a supervised overnight fast in 203 overweight and obese Latino children and adolescents (female/male: 88/115; mean age: 11.1 ± 1.7 years; pre-pubertal/pubertal/post-pubertal: 85/101/17; BMI z-score: 2.1 ± 0.4). Cumulative concentrations of NO_2,_ O_3_ and PM_2.5_ were spatially interpolated at the residential addresses based on measurements from community monitors up to 12 months prior to testing. Single and multi-pollutant linear effects models were used to test the cumulative monthly lag effects of NO_2_, O_3,_ and PM_2.5_ on morning serum cortisol levels after adjusting for age, sex, seasonality, social position, pubertal status, and body fat percent by DEXA.

**Results:**

Single and multi-pollutant models showed that higher O_3_ exposure (derived from maximum 8-h exposure windows) in the prior 1–7 months was associated with higher serum morning cortisol (*p* < 0.05) and longer term PM_2.5_ exposure (4–10 months) was associated with lower serum morning cortisol levels (*p* < 0.05). Stratification by pubertal status showed associations in pre-pubertal children compared to pubertal and post-pubertal children. Single, but not multi-pollutant, models showed that higher NO_2_ over the 4–10 month exposure period associated with lower morning serum cortisol (*p* < 0.05).

**Conclusions:**

Chronic ambient NO_2,_ O_3_ and PM_2.5_ differentially associate with HPA-axis dysfunction, a mechanism that may serve as an explanatory pathway in the relationship between ambient air pollution and metabolic health of youth living in polluted urban environments. Further research that uncovers how ambient air pollutants may differentially contribute to HPA-axis dysfunction are warranted.

**Supplementary Information:**

The online version contains supplementary material available at 10.1186/s12940-021-00713-2.

## Introduction

Elevated levels of circulating cortisol resulting from hypothalamic-pituitary-adrenal (HPA)-axis dysfunction have been associated with a variety of mental health [1–4], cardiovascular [5], and metabolic disorders, such as type 2 diabetes [6]. While causal models of HPA-axis dysregulation have been largely focused on either chronic psychosocial stressors [7–9], pre-existing clinical conditions (i.e., Cushing syndrome) [10], or fetal programming [11], recent experimental evidence suggests that exposure to air pollutants may play a role in central nervous system activation and may have downstream effects on HPA-axis activity, assessed via cortisol concentrations or diurnal patterning [12].

This emerging area of research has substantial evidence in animal models with respect to ozone (O_3_) and particulate matter (PM), which have been suggested to have extrapulmonary effects that may impact HPA-axis activity through indirect processes. For example, a possible mechanism of O_3_ effects on the brain may be attributed to vagal nerve activation by O_3_ gas thereby stimulating neural structures directly in the hypothalamus [13]. Other research suggests O_3_ and PM may have oxidative stress or inflammatory effects on the hippocampus and subsequent downstream effects on the hypothalamus and the HPA-axis [12]. To date, only a few clinical studies have reported associations between O_3_ or PM with altered HPA-axis activity. In a unique and seminal article in this field, researchers revealed that acute (2-h) O_3_ exposure in adults strikingly increased serum cortisol and corticosterone together with increases in lipid metabolism, 1-h post exposure [14]. With respect to the relationship between PM and HPA-axis function, results have been inconsistent with findings varying by PM source or size fraction and cortisol concentration or diurnal pattern. For example, one study revealed that participants exposed to higher indoor PM_2.5_ levels (9 days at an average of 53.1 μ g/m^3^) resulted in 1.3 mg/dL higher morning serum cortisol compared to those with real air purification (9 days at an average of 24.3 μ g/m^3^ [15]; however, other studies showed that higher exposure to concentrated ambient course PM was related to lower blood cortisol levels 1-h and 21-h post exposure [16], higher morning urinary cortisol [16], and higher cord blood cortisol in mothers [17]. Finally, there are two epidemiological studies which show that higher NO_2_ exposure over a 12-month period is associated with a blunted morning cortisol response [18, 19]. This experimental and epidemiological evidence suggests HPA-axis dysregulation from acute or chronic air pollution exposure, specifically as it pertains to NO_2_, O_3_ and PM_2.5_. This relatively small body of literature in humans on NO_2_, O_3_ and PM in relation to cortisol (measured in various biological tissues or matrices) is seemingly inconsistent, therefore more research is necessary to further establish and clarify these relationships, which may differ based by air pollutant. Moreover, the study of ambient air pollutant exposure during different temporal windows in relation to HPA-axis dysregulation in a high-risk youth population have remained unstudied.

It is of critical public health importance to understand underlying mechanisms associated with cardiometabolic health risk in the growing Latino population, which is also disproportionately affected by air pollution. It is estimated that by 2050, Latino peoples will compose over half of the California population [20]. Moreover, Latino people have a 50% lifetime risk of developing type 2 diabetes, the result of a complex mixture of predisposition, health behaviors, and environmental factors [21, 22]. For over two decades, reports show that Latino youth have the highest incidence and prevalence of obesity, prediabetes and type 2 diabetes in adults and is increasing among Latino adolescents during early to mid-adolescence [23, 24]. The HPA-axis has been shown to associate with type 2 diabetes risk in Latino youth [25, 26], and there is also evidence of high NO_2_ exposure contributing to a decline in the salivary morning cortisol response [19]; however, the role of air pollutant exposures on morning serum cortisol in a metabolically high risk Latino adolescent population has not been studied.

Based on the existing literature in rats and humans, the purpose of this secondary study was to investigate if 1-month to 12-month ambient NO_2_, O_3_, and PM_2.5_ exposures were associated with morning serum cortisol levels in overweight and obesity Latino youth with a family history of type 2 diabetes from the Los Angeles area. Utilizing a cross-sectional dataset, we hypothesized that cumulative exposures to ambient O_3_ and PM_2.5_ would be associated with altered serum morning cortisol levels, suggesting neuro-hormonal dysregulation of the HPA-axis. From a developmental standpoint, children and adolescents are more susceptible to psychological and pathological stressors during the pubertal process [27–29]. For these reasons, we explored if biological sex and pubertal stage confounded or modified the relationship between ambient air pollutants (NO_2_, O_3_, and PM_2.5_) and morning serum cortisol levels.

## Methods

### Participants

Analyses were conducted using baseline data of child participants aged 8–13 years from a cohort study known as the Study of Latino Adolescents at Risk (SOLAR), with the aim to investigate determinants of type 2 diabetes risk factors. A total of 203 overweight and obese (BMI percentile ≥ 85th percentile) Latino youth (88 female/115 male) from the greater Los Angeles region were included in this analysis who reported self, parents, and grandparents as Latino (or Hispanic). In addition to overweight/obesity status and Latino/Hispanic heritage, the presence of family history of type 2 diabetes was a final inclusion criterion, where at least one first-degree family member had been diagnosed with type 2 diabetes. Exclusion criteria included children with existing type 2 diabetes or those taking medications that may interfere with endocrine function or insulin sensitivity. Informed written consent from parents and child assent from the participant were obtained. The University of Southern California Institutional Review Board approved these studies. Details of the SOLAR full study protocol have been described previously [25, 26, 30–32].

### Brief description of study protocol

All participants attended two clinical visits (one outpatient and one inpatient) at the research unit of the Los Angeles County Hospital. The pertinent methods and procedures are described here with full study methodologies reported elsewhere [25, 26, 30–32]. During a full physical examination by a licensed practitioner and an oral glucose tolerance test, participants with results indicating type 2 diabetes diagnoses were immediately counseled by clinical staff and advised to follow-up with their pediatrician. These participants were then disqualified from the study, per study protocol. Approximately 2 weeks following the outpatient visit, qualified participants were admitted for an overnight inpatient visit at the same facility. Participants were given dinner and a snack and began fasting at 22:00 h, after which only water was permitted. At approximately 06:30 h the following morning, a 13-sampleinsulin-modified frequently sampled intravenous glucose tolerance test (FSIVGTT) was performed to model glucose and insulin metabolism dynamics (detailed methods described elsewhere) [31]. The two fasting blood samples that were taken at − 15 and − 5 min time points were later pooled and used for hormonal profiling, including the fasting morning serum cortisol concentrations.

### Assessment of HPA-axis function

Blood samples from the FSIVGTT were centrifuged for 10 min at 2500 rpm and serum aliquots were stored at − 80 °C. Morning serum cortisol concentrations were determined by radioimmunoassay kit (Siemens, Deerfield, Illinois); intra-assay CV 4.69%, inter-assay CV 6.28%, and minimal detection limit of 0.47 μg/dl.

### Residential air pollution exposure assessment

For ambient air pollutants (NO_2_, O_3_, and PM_2.5_), we assigned monthly air pollution exposure for up to 12 months prior to each participant visit. Street-level residential addresses of participants were geocoded at the parcel level (i.e., a building or lot of land) and match codes were output using the Texas A&M Geocoder (http://geoservices.tamu.edu/Services/Geocode/). When addresses did not initially match to a parcel centroid (e.g., address range interpolation or zip/city/state centroid) they were manually corrected using Google Maps based on the best available knowledge of the location of participant’s residence. Cases in which full street addresses were incomplete or not found were excluded from this analysis. For O_3_ specifically, we calculated monthly averages of daily maximum 8-h (hr) exposure (O_3_8-h max) and 24-h (O_3_24-h) concentrations. Exposures were spatially interpolated from the air quality monitoring station’s locations to the participant’s residence at the finest geographic resolution possible (usually parcel-level) using inverse distance-squared weighting. A correlation matrix of air pollutants exposures by month is shown in Supplemental Figure 1.

### Covariates

Pubertal maturation was determined by physical examination by a licensed health care provider using Tanner breast stage for girls [33] and Tanner pubic hair stage for boys [34], where those in Tanner 1 were classified to be in pre-puberty while those in Tanner 2–4 were classified mid-puberty, and Tanner 5 as post-puberty. Sex and date of birth (which was used to determine the average age between the outpatient and inpatient visits) were self-reported and later confirmed by a licensed health care provider. Total body fat percentage was determined at either visit by dual-energyx-ray absorptiometry using a Hologic QDR 4500 W (Hologic, Bedford, MA). The initial study design had no data on psychosocial stressors; therefore, we opted to utilize a broader social position variable to act as the best proxy for many psychosocial stressors that may be associated with socioeconomic/social position. To assess social position, we used the Hollingshead Four Factor Index of Social Status [35]. This index is a measure of a family’s social status which factors in the occupation (ascending scores from 1 to 9) and education (ascending scores of 1–7) of each parent/guardian residing in the child’s home. Individuals who reported being either homemakers, unemployed, or students (those who do not work outside the home, *n* = 123) did not fit into categories based on the Hollingshead method, therefore we employed a modified scoring system where these individuals were assigned a score of 0 in order to retain them in the final score (Supplemental Table 1). Education and occupation scores were weighted to obtain a single score for each parent/guardian and when there were multiple caretakers scores were averaged to obtain a single household social position score or index. Social position scores were then categorized into four categories (total *n* = 203): less than or equal to the 25th (*n* = 62), greater than the 25th percentile (*n* = 90) and less than the 75th, greater than or equal to the 75th percentile (*n* = 27), and missing education and occupational data (*n* = 24).

### Statistical analysis

Descriptive statistics (means and frequencies) of key variables were performed on the full sample and mean/frequency differences by pubertal status were performed with an analysis of variance (ANOVA) or chi-square where appropriate. Key variables were all normally distributed and did not require mathematical transformation. Multiple linear regression modeling was utilized to investigate unadjusted and adjusted associations with cumulative 1-month to 12-month average exposure to ambient air pollutants (24-h mean NO_2,_8-h maximum O_3_ [O_3_8-h max], 24-h mean O_3_ [O_3_24-h], and 24-h mean PM_2.5_). For each air pollutant, an unadjusted model was performed alongside a model adjusted for a priori covariates of age, pubertal status, sex, seasonality (warm/cold), total percent body fat, and social position, and an adjusted multi-pollutant model. Each multi-pollutant model was adjusted for each of the other pollutants for the same time cumulative monthly period. Effect modification by sex and pubertal status was also tested by adding a multiplicative interaction term, with a level of significance set at *p* < 0.1. All models passed when tested for linear model assumptions and model fit. Lastly, stratified analysis by pubertal status and sex were performed regardless of effect modification results given that there are known differences in HPA-axis activity by pubertal and sex status. Pollutant effect estimates were considered significant at a two-sided*p* < 0.05. All analyses were performed in SAS, version 9.4 (SAS, Institute, Cary, NC) and results are presented with unstandardized betas (using original scales), 95% confidence intervals (CI), and *p*-values.

## Results

Participants were between 8 and 13 years of age (mean: 11.1 and standard deviation: 1.7 years), 56% were female, and most were in pre-pubertal (40%) or mid-pubertal stage (52%), compared to only 7% who were post-pubertal stage. Across pubertal status, there were differences by weight, height, and BMI; however, there were no differences by age- and sex-adjusted BMI z-score or percent body fat. By design, most participants were in the obese category based on BMI classification (average BMI z-score of 2.1 ± 0.5). There were no differences in morning serum cortisol levels or by average 12-month exposure to NO_2_, O_3 _8-h, O_3 _24-h or PM_2.5_ across pubertal status (Table 1).

**Table 1:** Physical/Metabolic Characteristics and Ambient air Pollutant Exposure Averages of 203 Participants by Pubertal Status

	All (***n*** = 203) Mean (SD) or Frequency (%)	Pre- Puberty (***n*** = 85) Mean (SD) or Frequency (%)	Puberty (***n*** = 101) Mean (SD) or Frequency (%)	Post-Puberty (***n*** = 17) Mean (SD) or Frequency (%)	ANOVA ***P***-Value
**Physical/Metabolic Characteristics**					
Age (years)	11.1 (1.7)	10.0 (1.4)^a^	11.7 (1.4)^b^	13.2 (0.6)^c^	< 0.0001
Sex (n Female, % Female)	116 (56%)	62 (73%)	50 (50%)	4 (24%)	< 0.0001^q^
Weight (kg)	64.0 (19.7)	54.4 (15.7)^a^	68.4 (19.0)^b^	84.7 (17.6)^c^	< 0.0001
Height (cm)	148.7 (11.5)	141.3 (9.5)^a^	152.3 (9.6)^b^	162.7 (8.5)^c^	< 0.0001
BMI (kg/m^2^)	28.3 (5.5)	26.7 (5.0)^a^	29.0 (5.7)^b, c^	31.8 (4.8)^b, c^	< 0.0001
BMI z-score	2.1 (0.5)	2.1 (0.4)	2.0 (0.5)	2.1 (0.4)	0.89
Percent Body Fat (%)	38.4 (6.6)	38.8 (5.7)	38.5 (7.1)	36.6 (7.7)	0.27
Morning Serum Cortisol (mg/dL)	9.3 (3.2)	9.3 (2.9)	9.5 (3.4)	8.5 (3.2)	0.75
**Average 12-Month Exposures**					
NO_2_ (ppb)	32.8 (6.9)	33.3 (6.6)	32.5 (7.4)	31.8 (4.8)	0.51
O_3_ 8-Hour Maximum (ppb)	37.4 (13.6)	36.2 (14.2)	38.0 (13.6)	39.4 (10.5)	0.79
O_3_ 24-Hour (ppb)	20.9 (8.1)	20.3 (8.5)	21.2 (8.2)	21.7 (5.7)	0.6
PM_2.5_ (*μ*g/m^3^)	23.0 (6.2)	23.9 (6.9)	22.3 (5.7)	22.3 (6.1)	0.24

Different letters indicate group differences based on Bonferroni. ^q^Chi-Square *p*-value

*p < 0.05

**12-month averages

Among all Latino children, cumulative 1 to 7-month O_3 _8-h exposures were associated with higher morning serum cortisol levels before and after adjusting for potentially important confounders (age, sex, pubertal status, total body fat, season, and social position, Table 2). These relationships remained during the shorter term, cumulative 1 to 2-month and 4-month exposure windows in two-pollutant models that adjusted for NO_2_ and PM_2.5_, respectively (Table 2). For example, cumulative 1-month O_3_8-h exposure was associated with higher morning serum cortisol levels in the single pollutant model while adjusted for covariates (β = 0.07, 95% CI: 0.02, 0.12, *p* = 0.01) and the beta estimates remain largely unchanged in multi-pollutant models. Similarly, cumulative 2 to 7-month O_3 _24-h exposures were associated with higher morning serum cortisol levels after adjusting for potentially important confounders in single pollutant models (Table 3). In two-pollutant models, the effects were dampened in months 6–7; however, in months 1–5, the effects sizes stayed relatively the same despite loss of statistical significance. As an example, cumulative 2-month O_3_24-h exposure was associated with higher morning serum cortisol levels in the single pollutant model while adjusted for covariates (β = 0.10, 95% CI: 0.01, 0.09, *p* = 0.01) and the beta estimates remain largely unchanged in multi-pollutant models.

**Table 2:** Associations of Monthly Ambient O_3_ 8-h adjusted for NO_2_ and PM_2.5_ with Fasting Serum Cortisol in 203 Latino Children Enrolled in the SOLAR Study

	O_**3 **_24-h_**Max,**_ Unadjusted			O_**3 **_8-h_**Max,**_^a^			O_**3 **_8-h_**Max,**_ + NO_**2**_^a^			O_**3 **_8-h_**Max,**_ + PM_**2.5**_^a^		
Monthly Time Lag	β	95% CI	***p***-value	β	95% CI	***p***-value	β	95% CI	***p***-value	β	95% CI	***p***-value
1-month	0.05	0.01, 0.08	**0.01**	0.07	0.02, 0.12	**0.01**	0.07	0.01, 0.12	**0.02**	0.07	0.01, 0.12	**0.02**
2-month	0.05	0.02, 0.08	**0.003**	0.07	0.02, 0.11	**0.01**	0.06	0.01, 0.11	**0.02**	0.07	0.02, 0.12	**0.004**
3-month	0.05	0.02, 0.09	**0.003**	0.06	0.02, 0.1	**0.01**	0.05	−0.001, 0.1	0.05	0.06	0.02, 0.1	**0.01**
4-month	0.05	0.02, 0.09	**0.01**	0.05	0.01, 0.09	**0.01**	0.03	− 0.03, 0.09	0.3	0.04	0.003, 0.08	**0.04**
5-month	0.05	0.01, 0.09	**0.01**	0.05	0.01, 0.1	**0.01**	0.01	−0.5, 0.08	0.66	0.03	− 0.01, 0.08	0.14
6-month	0.06	0.01, 0.1	**0.02**	0.06	0.01, 0.1	**0.02**	0.01	−0.06, 0.08	0.82	0.03	− 0.02, 0.08	0.26
7-month	0.05	−0.001, 0.11	0.05	0.06	0.003, 0.12	**0.04**	0.01	−0.07, 0.09	0.83	0.03	−0.03, 0.09	0.32
8-month	0.05	− 0.01, 0.12	0.12	0.06	− 0.01, 0.13	0.08	0.01	−0.07, 0.09	0.8	0.03	−0.04, 0.1	0.43
9-month	0.04	−0.03, 0.12	0.25	0.05	−0.02, 0.13	0.16	0.01	−0.08, 0.10	0.84	0.03	−0.05, 0.1	0.51
10-month	0.04	−0.04, 0.12	0.3	0.05	−0.03, 0.13	0.25	0.01	−0.09, 0.10	0.87	0.03	−0.05, 0.11	0.49
11-month	0.05	−0.03, 0.13	0.23	0.05	−0.04, 0.13	0.26	0.02	−0.08, 0.11	0.74	0.04	−0.05, 0.12	0.39
12-month	0.07	−0.01, 0.15	0.1	0.06	−0.03 0.15	0.16	0.04	−0.06, 0.13	0.46	0.05	−0.03, 0.14	0.22

*NO*_*2*_ Nitrogen dioxide, *O*_*3*_ Ozone 8-h maximum daily, *PM*_2.5_ Particulate matter with aerodynamic diameter < 2.5 μm, *95% CI* 95% Confidence Interval; Results should be interpreted by original scaled variables

^a^Linear regression models adjusted for a priori covariates of age, sex, pubertal status, total body fat percentage, social position, season (warm/cool)

**Table 3:** Associations of Monthly Ambient O_3_ 24-h adjusted for NO_2_ and PM_2.5_ with Fasting Serum Cortisol in 203 Latino Children Enrolled in the SOLAR Study

	O_**3 **_24-h_**Max,**_ Unadjusted			O_**3 **_ 24-h^a^			O_**3 **_24-h + NO_**2**_^a^			O_**3 **_24-h + PM_**2.5**_^a^		
Monthly Time Lag	β	95% CI	***p***-value	β	95% CI	***p***-value	β	95% CI	***p***-value	β	95% CI	***p***-value
1-month	0.06	0.01, 0.11	**0.03**	0.08	−0.02, 0.17	0.11	0.07	−0.03, 0.18	0.18	0.07	−0.02, 0.17	0.13
2-month	0.07	0.02, 0.13	**0.01**	0.1	0.01, 0.09	**0.03**	0.1	− 0.01, 0.2	0.07	0.1	0.02, 0.19	**0.02**
3-month	0.08	0.02, 0.14	**0.01**	0.1	0.02, 0.17	**0.02**	0.08	− 0.03, 0.19	0.14	0.09	0.01, 0.17	**0.02**
4-month	0.09	0.02, 0.15	**0.01**	0.1	0.02, 0.17	**0.02**	0.04	− 0.08, 0.16	0.52	0.07	−0.001, 0.15	0.05
5-month	0.09	0.03, 0.16	**0.01**	0.1	0.02, 0.17	**0.01**	0.01	−0.12, 0.14	0.87	0.06	−0.02, 0.14	0.13
6-month	0.1	0.01, 0.19	**0.04**	0.1	0.02, 0.17	**0.01**	0.001	−0.14, 0.14	0.99	0.05	−0.03, 0.14	0.22
7-month	0.1	0.01, 0.19	**0.04**	0.1	0.01, 0.19	**0.04**	−0.001	−0.15, 0.14	0.98	0.06	−0.04, 0.15	0.25
8-month	0.09	− 0.01, 0.2	0.09	0.1	−0.01, 0.21	0.07	0.002	− 0.15, 0.16	0.98	0.06	−0.06, 0.17	0.32
9-month	0.08	−0.04, 0.21	0.12	0.09	−0.04, 0.22	0.15	−0.01	− 0.18, 0.17	0.95	0.06	−0.07, 0.19	0.36
10-month	0.08	−0.06, 0.22	0.27	0.08	−0.06, 0.23	0.26	−0.02	− 0.21, 0.17	0.83	0.06	−0.08, 0.21	0.4
11-month	0.09	−0.06, 0.23	0.25	0.08	−0.08, 0.23	0.32	− 0.01	− 0.21, 0.18	0.9	0.06	−0.09, 0.22	0.41
12-month	0.12	−0.03, 0.26	0.11	0.1	−0.06, 0.25	0.22	0.03	−0.17, 0.23	0.78	0.09	−0.07, 0.24	0.27

*NO*_*2*_ Nitrogen dioxide, *O*_*3*_ Ozone 8-h maximum daily, *PM*_*2.5*_ Particulate matter with aerodynamic diameter < 2.5 μm, *95% CI* 95% Confidence Interval; Results should be interpreted by original scaled variables

^a^Linear regression models adjusted for a priori covariates of age, sex, pubertal status, total body fat percentage, social position, season (warm/cool)

There was no evidence for effect modification by sex or pubertal status (data not shown, all *p* > 0.1). Based on known differences in pubertal development, we performed a stratified analysis by pubertal status (pre-puberty, puberty, or post-puberty stage). We observed stronger relationships in the pre-pubertal group only, compared to the later pubertal stages. For example, the single pollutant models show that cumulative 1-month O_3_8-h exposure was associated with higher morning serum cortisol levels in pre-pubertal youth (β = 0.13, 95% CI: 0.05, 0.21, *p* = 0.003), but not in mid-pubertal (β = 0.03, 95% CI: − 0.05, 0.11, *p* = 0.42) or post-pubertal youth (β = 0.04, 95% CI: − 0.28, 0.03, *p* = 0.77) after adjusting for key covariates covariates.

Single pollutant models for PM_2.5_ show longer term, cumulative 4 to 10-month PM_2.5_ exposures were associated with lower morning serum cortisol levels in adjusted models and while adjusting for covariates (Table 4). These relationships remained during the cumulative 5 to 9-month exposure windows in two-pollutant models after additional inclusion of NO_2_, O_3 _8-h or O_3 _24-h (Table 4). For example, cumulative 5-month PM_2.5_ exposure was associated with lower morning serum cortisol levels in the single pollutant model while adjusted for covariates (β = − 0.24, 95% CI: = − 0.39, − 0.10, *p* = 0.001) and the beta estimates slightly dampened but remained significant in multi-pollutant models. There was no evidence for effect modification by sex or pubertal status (data not shown, all *p* > 0.1). Similar to the aforementioned analyses with O_3 _8-h or O_3 _24-h on morning serum cortisol we performed a stratified analysis of these associations by pubertal status and observed stronger relationships in the pre-pubertal group only. For example, single pollutant models show that cumulative 4-month PM_2.5_ exposure was associated with lower morning serum cortisol levels in pre-pubertal youth (β = − 0.30, 95% CI: − 0.55, − 0.06, *p* = 0.02), but not in mid-pubertal (β = − 0.07, 95% CI: − 0.31, 0.17, *p* = 0.55) or post-pubertal youth (β = 0.03, 95% CI: − 0.86, 0.03, *p* = 0.91) after adjusting for key covariates.

**Table 4:** Associations of Monthly Ambient PM_2.5_ adjusted for NO_2_, O_3 _8-hr_Max_, and O_3 _24-h with Fasting Serum Cortisol in 203 Latino Children Enrolled in the SOLAR Study

	PM_**2.5**_ Unadjusted			PM_**2.5**_^a^			PM_**2.5**_ + NO_**2**_^a^			PM_**2.5**_ + O_**3 **_8-h_**Max**_^a^			PM_**2.5**_ + O_**3 **_24-h^a^		
Monthly Time Lag	β	95% CI	***p***-value	β	95% CI	***p***-value	β	95% CI	***p***-value	β	95% CI	***p***-value	β	95% CI	***p***-value
1-month	0.03	− 0.07, 0.13	0.58	0.04	−0.07, 0.15	0.46	0.07	−0.05, 0.18	0.24	0.02	−0.09, 0.13	0.75	0.03	−0.07, 0.14	0.54
2-month	−0.04	−0.14, 0.05	0.38	−0.03	− 0.15, 0.09	0.62	− 0.003	−0.13, 0.12	0.96	−0.06	− 0.18, 0.06	0.32	− 0.04	−0.16, 0.07	0.46
3-month	−0.1	−0.2, 0.01	0.08	−0.1	− 0.24, 0.04	0.15	− 0.06	− 0.21, 0.09	0.43	−0.1	− 0.23, 0.04	0.2	− 0.09	− 0.22, 0.04	0.19
4-month	−0.15	− 0.27, − 0.04	**0.01**	− 0.1	− 0.34, − 0.05	**0.01**	− 0.14	− 0.3, 0.03	0.1	− 0.16	− 0.31, − 0.02	**0.03**	− 0.16	− 0.31, − 0.02	**0.03**
5-month	− 0.19	− 0.31, − 0.08	**0.001**	− 0.24	− 0.39, − 0.1	**0.001**	− 0.18	− 0.35, − 0.02	**0.03**	− 0.21	− 0.36, − 0.05	**0.01**	− 0.21	− 0.36, − 0.06	**0.01**
6-month	− 0.22	− 0.35, − 0.1	**0.001**	− 0.26	− 0.41, − 0.11	**0.001**	− 0.2	− 0.37, − 0.03	**0.02**	− 0.23	− 0.38, − 0.07	**0.01**	− 0.23	− 0.38, − 0.07	**0.004**
7-month	− 0.25	− 0.39, − 0.1	**0.001**	− 0.27	− 0.43, − 0.12	**0.001**	− 0.21	− 0.4, − 0.03	**0.02**	− 0.24	− 0.41, − 0.08	**0.004**	− 0.24	− 0.41, − 0.08	**0.004**
8-month	− 0.3	− 0.46, − 0.12	**0.001**	− 0.32	− 0.5, − 0.13	**0.001**	− 0.26	− 0.47, − 0.05	**0.02**	− 0.29	− 0.49, − 0.1	**0.004**	− 0.29	− 0.48, − 0.1	**0.003**
9-month	− 0.3	− 0.5, − 0.1	**0.003**	− 0.31	− 0.52, − 0.1	**0.004**	− 0.25	− 0.48, − 0.02	**0.04**	− 0.29	**−** 0.51, − 0.07	**0.01**	− 0.29	− 0.5, − 0.08	**0.01**
10-month	− 0.27	− 0.47, − 0.06	**0.01**	− 0.26	− 0.48, − 0.04	**0.02**	− 0.2	− 0.44, 0.04	0.11	− 0.25	− 0.47, − 0.02	**0.03**	− 0.25	− 0.47, − 0.03	**0.03**
11-month	− 0.19	− 0.4, 0.02	0.08	− 0.18	− 0.41, 0.04	0.11	− 0.2	− 0.37, 0.13	0.35	− 0.17	− 0.39, 0.06	0.15	− 0.17	− 0.4, 0.05	0.13
12-month	− 0.17	− 0.38, 0.04	0.12	− 0.15	− 0.38, 0.08	0.2	− 0.08	− 0.33, 0.18	0.54	− 0.13	− 0.36, 0.1	0.26	− 0.14	− 0.36, 0.09	0.24

*NO*_*2*_ Nitrogen dioxide, *O*_*3*_ Ozone 8-h maximum daily, *PM*_*2.5*_ Particulate matter with aerodynamic diameter < 2.5 μm, *95% CI* 95% Confidence Interval; Results should be interpreted by original scaled variables

^a^Linear regression models adjusted for a priori covariates of age, sex, pubertal status, total body fat percentage, social position, season (warm/cool)

Single pollutant models for NO_2_ show longer term, cumulative 4 to 10-month NO_2_ exposures were associated with lower morning serum cortisol levels in unadjusted models and adjusting for covariates, however these results were not significant in multi-pollutant models (Table 5). For example, cumulative 4-month NO_2_ exposure was associated with lower morning serum cortisol levels in the single pollutant model while adjusted for covariates (β = − 0.13, 95% CI: − 0.23, − 0.03, *p* = 0.009) and the beta estimates dampened and no longer significant in multi-pollutant models. There was no evidence for effect modification by sex or pubertal status (data not shown, all *p* > 0.1). The stratified analysis of these associations shows a stronger relationship in the pre-pubertal group. For example, cumulative 4-month NO_2_ exposure was associated with lower morning serum cortisol levels in pre-pubertal youth (β = − 0.22, 95% CI: − 0.37, − 0.07, *p* = 0.004), but not in mid-pubertal (β = − 0.03, 95% CI: − 0.17, 0.12, *p* = 0.71) or post-pubertal youth (β = − 0.36, 95% CI: − 0.98, 0.25, *p* = 0.21) after adjusting for previously mentioned covariates.

**Table 5:** Associations of Monthly Ambient NO_2_ adjusted for PM_2.5_, O_3_-8hr_Max_, and O_3_-24h with Fasting Serum Cortisol in 203 Latino Children Enrolled in the SOLAR Study

	NO_**2**_ Unadjusted			NO_**2**_^**a**^			NO_**2**_+ PM_**2.5**_^**a**^			NO_**2**_ + O_**3 **_8-h_**Max**_^**a**^			NO_**2**_+ O_**3 **_24-h^**a**^		
Monthly Time Lag	β	95% CI	***p***-value	β	95% CI	***p***-value	β	95% CI	***p***-value	β	95% CI	***p***-value	β	95% CI	***p***-value
1-month	− 0.06	− 0.13, 0.01	0.11	− 0.04	− 0.14, 0.06	0.39	− 0.07	− 0.18, 0.04	0.21	− 0.01	− 0.12, 0.09	0.8	− 0.01	−0.12, 0.11	0.91
2-month	−0.07	−0.14, 0.004	0.06	−0.07	− 0.17, 0.03	0.19	− 0.07	−0.18, 0.04	0.23	−0.02	− 0.13, 0.1	0.78	− 0.01	−0.13, 0.12	0.93
3-month	−0.09	−0.16, − 0.01	**0.02**	−0.1	− 0.2, 0.002	0.06	− 0.08	−0.19, 0.03	0.14	−0.03	− 0.15, 0.1	0.66	− 0.03	−0.16, 0.11	0.72
4-month	−0.11	−0.19, − 0.33	**0.01**	−0.13	− 0.23, − 0.03	**0.01**	−0.09	− 0.12, 0.02	0.1	− 0.08	−0.22, 0.06	0.25	−0.09	− 0.25, 0.07	0.25
5-month	−0.13	−0.21, − 0.05	**0.002**	−0.14	− 0.24, − 0.05	**0.004**	−0.08	− 0.19, 0.03	0.13	− 0.12	−0.26, 0.03	0.11	−0.13	− 0.3, 0.03	0.12
6-month	−0.14	−0.24, − 0.05	**0.002**	−0.15	− 0.25, − 0.05	**0.004**	−0.08	− 0.12, 0.04	0.2	− 0.14	−0.29, 0.01	0.07	−0.15	− 0.31, 0.02	0.08
7-month	−0.15	−0.25, − 0.05	**0.004**	−0.15	− 0.25, − 0.04	**0.01**	−0.07	− 0.2, 0.05	0.23	− 0.14	−0.28, 0.01	0.06	−0.15	− 0.31, 0.01	0.07
8-month	−0.15	−0.25, − 0.04	**0.01**	−0.15	− 0.26, − 0.03	**0.01**	−0.07	− 0.2, 0.06	0.3	− 0.14	−0.28, 0.01	0.07	−0.15	− 0.31, 0.01	0.07
9-month	−0.14	−0.26, − 0.02	**0.02**	−0.14	− 0.26, − 0.02	**0.02**	−0.08	− 0.2, 0.06	0.26	− 0.13	−0.28, 0.01	0.07	−0.14	− 0.31, 0.02	0.08
10-month	−0.14	−0.26, − 0.01	0.03	−0.14	− 0.27, − 0.01	**0.04**	−0.09	− 0.23, 0.06	0.23	− 0.13	−0.28, 0.02	0.08	−0.15	− 0.31, 0.01	**0.02**
11-month	−0.12	−0.25, 0.01	0.07	−0.12	− 0.25, 0.02	0.08	− 0.09	−0.23, 0.06	0.25	−0.11	− 0.25, 0.05	0.17	− 0.12	−0.29, 0.05	0.15
12-month	−0.12	−0.25, 0.01	0.07	−0.11	− 0.24, 0.02	0.11	− 0.09	−0.24, 0.06	0.25	−0.08	− 0.24, 0.07	0.27	− 0.09	−0.27, 0.08	0.28

*NO*_*2*_ Nitrogen dioxide, *O*_*3*_ Ozone 8-h maximum daily, *PM*_*2.5*_ Particulate matter with aerodynamic diameter < 2.5 μm, *95% CI* 95% Confidence Interval; Results should be interpreted by original scaled variables

^**a**^Linear regression models adjusted for a priori covariates of age, sex, pubertal status, total body fat percentage, social position, season (warm/cool)

## Discussion

Results of this cross-sectional study show that shorter-term, chronic (1–7 month) O_3_ exposure was associated with higher circulating serum cortisol levels while longer-term (4–10 month) NO_2_ and PM_2.5_ exposure was associated with lower serum cortisol in overweight and obese Latino youth, while adjusting for covariates. Furthermore, effect sizes of the O_3_ and PM_2.5_ models were largely unchanged in multi-pollutant models. Our findings are partially supported by the literature that show: 1) experimental studies that suggest HPA-axis alterations may result from acute O_3_ and PM_2.5_ exposures and 2) observational studies in humans which show associations between chronic NO_2_ and dampened morning cortisol responses.

There is mounting biological and epidemiological evidence suggesting that the physical environment plays a role in the rise of mental health issues [1–4] and metabolic diseases worldwide [6], however further consideration of the intermediary mechanisms, such as HPA-axis dysregulation has been understudied. To date, consideration of an environmental-neurobiological approach and its effects on HPA-axis dysregulation is still in its infancy. Prior knowledge on HPA-axis dysregulation have been largely focused on either chronic psychosocial stressors, sleep patterns, pre-existing clinical conditions (i.e., Cushing syndrome), or fetal programming [11]. Our study, along with several others, considers that chronic exposure to air pollutants, such as ambient NO_2_, O_3_ and PM_2.5_, have extra-pulmonary, neurohormonal effects that may impact the hypothalamus and possibly other brain structures, which then culminate in HPA-axis dysregulation. Our results show some evidence for this proposed causal pathway in which higher O_3_ was associated with a higher morning cortisol while higher NO_2_ and PM_2.5_ were associated with lower morning cortisol concentrations.

There is strong evidence in experimental models from the past decade that air pollutants (as acute, concentrated exposures) can act as stressors that may result in higher circulating corticoid levels. For example, acute ozone (O_3_) exposure in rats increased production of corticosterone [36, 37] which is the counterpart to cortisol in humans. A separate study showed that exposing rats to concentrated ambient particles activated brain stress centers and increased circulating levels of corticosterone [38]. Another study in rats showed that acute exposure to O_3_ or PM_2.5_ increased blood levels of ACTH (a corticosterone precursor) and corticosterone, suggesting that single exposures to either O_3_ or PM_2.5_ can activate the HPA-axis [39]. In rats, O_3_ exposure showed a brain activated pattern that may suggest signaling via the vagus nerve [13]. Another potential mechanism that has been proposed is glucocorticoid-regulated gene alteration in rodent brains and subsequent increases in glucocorticoid concentrations in the brain [12]. In conjunction with the neurotoxicity hypothesis [40], which suggests hippocampal atrophy following cumulative glucocorticoid exposure in the brain, there is some evidence to support dysregulation of HPA-axis activity [12], given the regulatory role of the hippocampus on the HPA-axis presumably during sleep and the pre-awakening phase [41]. Yet another potential mechanism suggests that O_3_ reacts with pulmonary lipids to produce secondary products released in the systemic circulation [42]. Collectively, these initial studies suggest possible mechanisms linking O_3_ and PM_2.5_ to HPA-axis dysregulation in the form of higher glucocorticoids.

In the current study, 1–7 month ambient O_3_ exposure was associated with higher morning cortisol, findings which contrast with the association between 4 and 10 month exposure to PM_2.5_ with lower morning cortisol. The results with O_3_ and morning cortisol are in line with the experimental evidence that have tested the effects of acute exposure to O_3_ and PM in controlled environments. First, in a panel study, controlled exposure to 0.3 ppm O_3_ for 2 h increased blood stress hormone levels, including cortisol, one-hour post exposure [14]. Another study which considered larger PM particles revealed distinct findings, showing that 130-min exposure to high levels of coarse concentrated ambient particles (sized 2.5–10 μm; mean 238 *μ*g/m^3^) was related to lower blood cortisol levels [16]. Both of these show support for our findings, yet there are also contradictory findings with relation to PM_2.5_ exposures. In a randomized double-blind crossover design study, higher nine-day average indoor PM_2.5_ exposure (average of 53.1 μg/m^3^) was associated with higher morning plasma cortisol levels compared to those exposed to lower PM_2.5_ with use of an air purifier (average of 24.3 μg/m^3^) [15]. To date, there has been no clear epidemiological evidence that chronic exposure to ambient O_3_ and PM associates with HPA-axis activity. This study may be the first to show these relationships.

Our results that show a relationship between higher NO_2_ exposures and lower morning serum cortisol supplement the current observational studies in the field. First, in a nationally representative sample of 1793 middle aged to elderly adults (45–85 years, 56% Latino, mean BMI: 29 kg/m^2^), 5-year longitudinal results showed that for every 10 ppb increase of NO_2_ there was a 0.014 increase in the diurnal slope (wake-to-bedtime), which indicates a more blunted response over time in response the higher NO_2_ exposure [18]. Second, in a sample of 140 adolescents (mean age: 14 years, 69% Latino, mean BMI: 24 kg/m^2^), those exposed to the estimated 75th percentile of ambient NO_2_ (high exposure) exhibited a more blunted cortisol awakening response (CAR; the difference in cortisol from awakening to 30 min post awakening) relative to those exposed at the 25th percentile of ambient NO_2_ (low exposure) [19]. This limited literature evaluates the outcome using salivary measures to plot the diurnal cortisol responses that represent the dynamic function of the HPA-axis, compared to our morning serum measure which represents the basal HPA-axis activity; therefore, it is challenging to make any direct comparisons with our results. Our findings may be reflective of pre-awakening neurohormonal mechanisms, rather than the dynamic cortisol response from pre- to post-awakening. Specifically, it has been shown that extra-pituitary mechanisms influence HPA-axis activity differently prior to awakening and post-awakening, which may in part explain our findings [41]. During sleep and prior to awakening, decreased HPA sensitivity to slow or “fine tune” the release of cortisol is governed by the hippocampus, suprachiasmatic nucleus, and sympathetic nervous system [41]. Upon awakening, changes in brain region activation result in increased HPA-axis sensitivity and a rapid elevation in cortisol with attainment of consciousness [41]. For these reasons, the measurement of basal levels of cortisol and the CAR may represent separate parameters governed by different parts of the brain. As previously mentioned, direct comparisons of our findings and the literature cannot be made due to the differences in HPA-axis assessment. Our results show that both higher exposure to NO_2_ and PM_2.5_ associated with lower morning serum cortisol, which may suggest a more blunted CAR, but we cannot assert this statement based on the scope of this study.

The importance of future research in this field cannot be understated. The findings of the current study are novel as there are no experimental, clinical, or epidemiological studies which show opposing directions in the relationships between various air pollutants and a measure of cortisol. We speculate that the high complexity of the HPA-axis by way of multiple feedback loops and their different governing mechanisms in the brain may suggest that air pollutants may differentially impact HPA-dysfunction in the form of alterations to either basal morning cortisol concentrations or by exerting changes to the dynamic CAR [41]. Our morning serum cortisol measure was not timed or repeated to fully capture these differences. Despite the limited availability of literature in this field, there is sufficient evidence to suspect that ambient air pollutants may play a role in HPA-axis dysfunction, in the form of either heightened or dampened cortisol basal concentration or dynamic responses. Mechanistic studies and well-designed epidemiological studies which address key confounders will be an important consideration for future research.

In this study of children, special consideration of pubertal maturation and associated sex hormones effects on HPA-axis activity was required. During this time, the brain undergoes a reorganization process known as synaptic pruning and increased brain plasticity, which may also be impacted by rise of sex hormones that may further affect neural networks such as those related to HPA-axis activity [43]. The results of our study showed differences by sex or pubertal status were not observed via statistical interactions, however based on known differences in pubertal development, we performed a stratified analysis by sex and pubertal status. Our results shown stronger relationships with O_3 _8-h and PM_2.5_ and fasting blood cortisol in the prepubertal youth compared to the mid-pubertal and post-pubertal groups. Despite these relationships that were independent of sex, the timing of menstrual cycles with the blood draw collection may have an impact on HPA-axis activity and cortisol production [44]. It was outside the scope of the study design to adjust for menstrual cycle relative to the blood collection, therefore any cortisol variation in females during mid-puberty and post-puberty may have attenuated effect between air pollution and basal cortisol levels. Further study would be necessary to take into account differences by sex and pubertal status.

HPA-axis dysregulation may be a key mediating mechanism linking air pollution and neurological and metabolic health of children living in polluted urban environments. First, there is ample support showing the associations of NO_2_, O_3_ and PM_2.5_ exposure with a variety of central nervous system and metabolic health impacts. These include cognitive function [45], anxiety [46, 47] and depression [1, 2, 4, 48], and type 2 diabetes risk [30, 32]. Next, various components of HPA-axis dysregulation have also been linked to mental health or metabolic outcomes. As an example, lower cortisol levels have been shown to be associated with recurrence of depression in middle-aged adults [49] and with disruptive psychopathology in adolescents [50]. With respect to metabolic health outcomes in Latino youth, higher morning serum cortisol at baseline and a 2-year increase in cortisol was associated with type 2 diabetes risk factors, including higher fasting glucose and lower insulin sensitivity [25, 26]. These studies suggest that HPA-axis activity may play a mediating or moderating role in the relationship between air pollution and type 2 diabetes risk factors in Latino overweight and obese youth. Collectively, this body of evidence allows us to more seriously consider HPA-axis dysregulation as a key mediating mechanism between the effect of air pollution exposure and a variety of mental health and metabolic disease outcomes.

Strengths of this study include the rigorous methods of the clinically relevant variables and the population of high-risk children. First, the morning serum cortisol was assayed from a fasting blood draw that was collected in an inpatient setting following a supervised overnight fast. Measurement of cortisol in the morning reflects basal levels of HPA-axis activity prior to any daily acute stressors which may impact short term cortisol concentrations. Key covariates such as total body fat composition was determined by dual x-ray absorptiometry (DEXA; the gold standard in body composition measurement), pubertal staging and medical history intake were assessed by a licensed clinician. With relationship to our sample population, our study’s sampling strategy focused on mostly lower social position, overweight and obese Latino children with a family history of type 2 diabetes. Although our target population is narrow, our results may be generalizable to a rapidly growing segment of urban U.S. Latino population thereby increasing the importance of public health prevention measures. For example, in the region of greater Los Angeles, our source population, the current proportion of Latino youth stands at 55.7% (U.S. Census, July 2020). Our results could be extended to all populations of children living in urban regions, particularly those who may have other risk factors related to mental health such as depression, pediatric obesity, or/and family history of metabolic disease.

This study also has several limitations associated with the original, clinically based study design, with the aim of studying the role of fat deposition and metabolic syndrome on type 2 diabetes risk factors in childhood. First, residential mobility in the year prior to study enrollment was not assessed, nor were various acute or chronic stress measures (psychometrics and HPA-axis function assessment) as these methods were out of the scope of the original study aims. Specifically, psychosocial stressors [7–9] or sleep quality/patterns [51], which are well known to contribute to HPA-dysfunction, were not assessed in the study. For this reason, we were unable to assess if our findings were independent of important known psychosocial confounders. Similarly, our original study aims did not address neighborhood contextual variables, such as neighborhood level socioeconomic status, noise contamination, or violence, all of which could be possible confounders of findings. Our best attempt to adjust for these was the use of our social position variable, which is a good proxy for many of the key neighborhood contextual variables.

With regard to our HPA-axis assessment methodology, there are considerations to acknowledge on the single morning serum cortisol measure. A single measure may not be sufficient to show chronic HPA-axisdysfunction, such as hyper- or hypo-cortisolism. Furthermore, we cannot exclude the possibility of confounding by more proximal events (personal life or neighborhood stressors), to the time of cortisol assessment. For these reasons, an average of repeated serum or saliva morning measures over many days or a cumulative measure of cortisol (such as that reflected in a sample of hair [52],) would provide a more accurate assessment of HPA-axis function during air pollution exposure windows. On the other hand, there is ample clinical evidence to suggest that a single morning measure may be used to indicate HPA axis dysregulation that associates with cardiometabolic diseases. First, serum cortisol can be clinically useful and correlates well with salivary cortisol in diagnosing adrenal disorders that are characterized by chronic HPA under- or over-activity, such as Addison’s disease and Cushing’s syndrome [53–55]. Second, evidence from clinical and epidemiological studies have associated higher basal HPA-axis activity, by way of a single morning measure, with various chronic illnesses such as cardiovascular disease [56], type 2 diabetes [57], as well as structural changes in the brain [58]. In order to determine whether longer term air pollution truly plays a role in HPA axis function, future study methodologies should include repeated measures or longer-term markers of HPA-axis activity.

Another limitation is the cross-sectional design and the uneven distribution of participants at each of the five Tanner stages where samples sizes in children at Tanner stages 4 and 5 were low. In addition, the distribution of sex by pubertal stage was also unequal and did not provided sufficient power to conduct a three-wayinteraction. Next, a more reliable variable that better represented the socio-economic status construct, and instead we used a social position variable that did not include income. A few limitations exist with respect to the air pollution exposure data. Residential based estimates of air pollution exposure may have resulted in exposure misclassification since they do not account for personal time-activity or behavioral factors that could affect personal exposures; however, others have shown that this type of misclassification likely weakens statistical power towards the null [59]. Finally, it known that short term variation in temperature may impact air pollution exposure and possibly even cortisol levels, however measurement of these fluctuations was out of the scope of our study. Our best proxy to address this issue was to adjust for seasonality. A future environmental epidemiological study in children with a primary aim of addressing ambient air pollution as a determinant of HPA-axis activity which addresses all of these vital confounders would be necessary.

In conclusion, our results suggest that NO_2_, O_3_ and PM_2.5_ may differentially influence HPA-axis activity in distinctive exposure windows in metabolically high-risk, Latino youth. Specifically, shorter term O_3_ exposure was associated with higher circulating serum cortisol levels; however, longer term NO_2_ and PM_2.5_ exposure was associated with lower serum cortisol. These results suggest that chronic exposure to ambient air pollutants play a role in the HPA-axis function, possibly via alterations in brain physiology. Future studies that incorporate the study of cortisol precursors, a wider variety of biospecimens and techniques for cortisol profiling, and personal exposure histories are warranted.

## Supplementary Information

**Additional file 1: Supplemental Table 1.** Baseline Social Position Information in Exposure in Latino Children Enrolled in the Longitudinal Study for Those with Social Position Information. **Figure 1.** Correlation Matrix of Monthly Ambient Air Pollutants (NO_2_, O_3_, and PM_2.5_).

## Data Availability

The data that support the study findings are restricted for transmission to those outside the primary SOLAR investigative team. Data sharing with investigators outside the SOLAR team requires IRB approval. Requests may be submitted to the corresponding author.
