# Supplementary material for: Ambient air pollutants are associated with morning serum cortisol in overweight and obese Latino youth in Los Angeles
Source: Environ Health. 2021 Apr 8;20:39. doi: 10.1186/s12940-021-00713-2 (PMC8034084; doi:10.1186/s12940-021-00713-2)
Supplement: Supplementary file 1 — Additional file 1: Supplemental Table 1. Baseline Social Position Information in Exposure in Latino Children Enrolled in the Longitudinal Study for Those with Social Position Information. Figure 1. Correlation Matrix of Monthly Ambient Air Pollutants (NO2, O3, and PM2.5). [file 12940_2021_713_MOESM1_ESM.docx]

**Supplemental Table 1. Baseline Social Position Information in Exposure in Latino Children Enrolled in the Longitudinal Study for Those with Social Position Information**

| **Social Position** | **All (n=179)** | **Pre- Puberty**  **(n=76)** | **Puberty**  **(n=89)** | **Post-Puberty (n=14)** | **P-Value** |  |  |  |  |
| --- | --- | --- | --- | --- | --- | --- | --- | --- | --- |
| Modified Four Factor Index Score | 16.49 ± 9.26 | 16.59. ± 9.96 | 16.69 ± 9.25 | 14.68 ± 4.38 | 0.75^1^ |  |  |  |  |
| **Marital Status** |  |  |  |  | 0.24^2^ |  |  |  |  |
| Single | 23 (13%) | 7 (9%) | 16 (18%) | 0 (0%) |  |  |  |  |  |
| Married / Civil Union | 128 (72%) | 54 (71%) | 61 (69%) | 13 (93%) |  |  |  |  |  |
| Living Together | 2 (1%) | 1 (1%) | 1 (1%) | 0 (0%) |  |  |  |  |  |
| Divorced / Separated | 24 (13%) | 14 (19%) | 9 (10%) | 1 (7%) |  |  |  |  |  |
| Widow | 2 (1%) | 0 (0%) | 2 (2%) | 0 (0%) |  |  |  |  |  |
| **Mom Education**^a^ **(N=177)** |  |  |  |  | 0.34^2^ |  |  |  |  |
| <7^th^ Grade | 62 (35%) | 26 (35%) | 31 (35%) | 5 (36%) |  |  |  |  |  |
| 7-9^th^ Grade | 38 (22%) | 15 (20%) | 16 (18%) | 7 (50%) |  |  |  |  |  |
| Some High School | 21 (12%) | 10 (13%) | 11 (13%) | 0 (0%) |  |  |  |  |  |
| GED | 36 (20%) | 15 (20%) | 19 (22%) | 2 (14%) |  |  |  |  |  |
| Some University | 315 (8%) | 9 (12%) | 6 (7%) | 0 (0%) |  |  |  |  |  |
| Bachelors Degree | 3 (2%) | 0 (0%) | 3 (3%) | 0 (0%) |  |  |  |  |  |
| Graduate School | 2 (1%) | 0 (0%) | 2 (2%) | 0 (0%) |  |  |  |  |  |
| **Dad Education**^b^ (N=131) |  |  |  |  | 0.69^2^ |  |  |  |  |
| <7^th^ Grade | 48 (37%) | 21 (37%) | 21 (34%) | 6 (46%) |  |  |  |  |  |
| 7-9^th^ Grade | 33 (25%) | 13 (23%) | 19 (31%) | 1 (8%) |  |  |  |  |  |
| Some High School | 17 (13%) | 9 (16%) | 6 (10%) | 2 (15%) |  |  |  |  |  |
| GED | 23 (18%) | 9 (16%) | 12 (20%) | 2 (15%) |  |  |  |  |  |
| Some University | 6 (5%) | 3 (5%) | 2 (3%) | 1 (8%) |  |  |  |  |  |
| Bachelors Degree | 4 (3%) | 2 (3%) | 1 (2%) | 1 (8%) |  |  |  |  |  |
| Graduate School | 0 (0%) | 0 (0%) | 0 (0%) | 0 (0%) |  |  |  |  |  |
| **Mom Occupation**^a^ (N=176) |  |  |  |  | 0.62^2^ |  |  |  |  |
| Does Not Work Outside the Home | 111 (63%) | 41 (63%) | 53 (61%) | 11 (79%) |  |  |  |  |  |
| Farm Laborers / Menial Service Workers | 14 (8%) | 6 (8%) | 8 (9%) | 0 (0%) |  |  |  |  |  |
| Unskilled Workers | 18 (10%) | 9 (12%) | 7 (8%) | 2 (14%) |  |  |  |  |  |
| Machine Operators and Semiskilled Workers | 11 (6%) | 5 (7%) | 6 (7%) | 0 (0%) |  |  |  |  |  |
| Skilled Manual Workers, Craftsman | 9 (5%) | 1 (1%) | 8 (9%) | 0 (0%) |  |  |  |  |  |
| Clerical and Sales Workers, Small Business Owners | 6 (4%) | 2 (3%) | 3 (4%) | 1 (7%) |  |  |  |  |  |
| Technicians, Semiprofessionals, Small Business Owners | 5 (3%) | 3 (4%) | 2 (2%) | 0 (0%) |  |  |  |  |  |
| Smaller Business Owners, Minor Professionals | 1 (0.5%) | 1 (1%) | 0 (0%) | 0 (0%) |  |  |  |  |  |
| Administrators, Lesser Professionals | 1 (0.5%) | 1 (1%) | 0 (0%) | 0 (0%) |  |  |  |  |  |
| Higher Executives, Major Professionals | 0 (0%) | 0 (0%) | 0 (0%) | 0 (0%) |  |  |  |  |  |
| **Dad Occupation^c^ (N=129)** |  |  |  |  | 0.91^2^ |  |  |  |  |
| Does Not Work Outside the Home | 12 (9%) | 4 (7%) | 6 (10%) | 2 (15%) |  |  |  |  |  |
| Farm Laborers / Menial Service Workers | 1 (1%) | 0 (0%) | 1 (2%) | 0 (0%) |  |  |  |  |  |
| Unskilled Workers | 32 (25%) | 18 (31%) | 12 (21%) | 2 (15%) |  |  |  |  |  |
| Machine Operators and Semiskilled Workers | 52 (40%) | 21 (36%) | 25 (43%) | 6 (47%) |  |  |  |  |  |
| Skilled Manual Workers, Craftsman | 29 (23%) | 13 (22%) | 13 (22%) | 3 (23%) |  |  |  |  |  |
| Clerical and Sales Workers, Small Business Owners | 1 (2%) | 1 (2%) | 1 (2%) | 0 (0%) |  |  |  |  |  |
| Technicians, Semiprofessionals, Small Business Owners | 1 (1%) | 1 (2%) | 0 (0%) | 0 (0%) |  |  |  |  |  |
| Smaller Business Owners, Minor Professionals | 0 (0%) | 0 (0%) | 0 (0%) | 0 (0%) |  |  |  |  |  |
| Administrators, Lesser Professionals | 0 (0%) | 0 (0%) | 0 (0%) | 0 (0%) |  |  |  |  |  |
| Higher Executives, Major Professionals | 0 (0%) | 0 (0%) | 0 (0%) | 0 (0%) |  |  |  |  |  |

Baseline social position characteristics in Latino youth. Data are reported as mean with standard deviation (SD) or with sample number and percent. Sample size is indicated as ^a^177, ^b^131, and ^c^129. Education and occupations are shown in order of ascending scores from 1-7 and 0-9, respectively. ^1^ANOVA ^2^Fisher’s Exact Test

**Figure 1: Correlation Matrix of Monthly Ambient Air Pollutants (NO_2_, O_3_, and PM_2.5_)**
